# Comparative Evaluation of the Effectiveness of Transcutaneous Electrical Nerve Stimulation (TENS) and Low-Level Laser Therapy (LLLT) in Symptomatic Temporomandibular Disorders: A Randomised Controlled Trial

**DOI:** 10.7759/cureus.108391

**Published:** 2026-05-06

**Authors:** Debasree Boral, Kanad Chaudhuri, Sayan Chattopadhyay, Rritam Ghosh, Debanti Giri, Keerthi K Nair, Rachita Arora

**Affiliations:** 1 Oral Medicine and Radiology, Dr. R. Ahmed Dental College and Hospital, Kolkata, IND; 2 Oral Medicine and Radiology, JIS School of Medical Science and Research, Kolkata, IND; 3 Pediatric and Preventive Dentistry, Dr. R. Ahmed Dental College and Hospital, Kolkata, IND

**Keywords:** diode laser, jaw function, low-level laser therapy, pain management, temporomandibular joint disorders, transcutaneous electrical nerve stimulation

## Abstract

Background: A common musculoskeletal ailment affecting the temporomandibular joint (TMJ) and masticatory muscles, temporomandibular disorders (TMDs) can cause discomfort and limited jaw mobility. Low-level laser therapy (LLLT) and transcutaneous electrical nerve stimulation (TENS) are two non-invasive therapeutic techniques that have shown promise in improving function and reducing pain. However, comparative data about their efficacy are still limited. This randomised controlled experiment aimed to assess and contrast how well TENS and LLLT work to improve maximum mouth opening (MMO) and lessen discomfort in individuals with TMD.

Methods: A randomised clinical trial was conducted at Dr. R. Ahmed Dental College and Hospital, Kolkata, on 40 patients with symptomatic TMDs. Patients were randomly assigned into two groups (n=20 per group): Group A received TENS therapy, and Group B underwent LLLT for eight sessions. Pain levels were assessed using a visual analogue scale (VAS), and MMO was measured at baseline, mid-treatment, and post-treatment. Statistical analyses included repeated measures ANOVA and unpaired t-tests, with significance at P<0.05.

Results: Both groups exhibited a significant reduction in pain scores and increased MMO (P<0.001). However, LLLT demonstrated a significantly greater reduction in pain at multiple time points compared to TENS (P=0.004). Additionally, LLLT resulted in a more pronounced MMO increase, particularly at mid-treatment (P=0.006).

Conclusion: TENS and LLLT are effective for TMD management, but LLLT provides superior pain relief and functional improvement. Further research is recommended to explore their long-term efficacy and combined therapeutic potential.

## Introduction

Musculoskeletal disorders known as temporomandibular joint disorders (TMDs) impact the masticatory system, which includes the temporomandibular joint (TMJ), the masticatory muscles, and related head and neck structures [1]. These disorders are highly prevalent, affecting approximately 5-12% of the adult population [2].

Research indicates that 40-75% of asymptomatic individuals exhibit at least one clinical sign of TMD, while nearly one-third report experiencing at least one symptom [1,2]. Although TMDs can occur at any age, they are most observed in individuals between 20 and 40 years of age [3]. Furthermore, epidemiological data suggest that women are affected at a rate two to four times higher than men [4].

The genesis of TMDs is complex and multifactorial, and the disorder presents with a wide spectrum of clinical manifestations. The principal symptoms and signs include masticatory pain, headaches, limited mandibular movement with deviation, temporomandibular joint sounds, subluxation or jaw locking, and functional limitations during mastication or speech [2]. Patients may also report associated complaints such as neck pain, sleep disturbances, tinnitus, and preauricular discomfort [2,5]. In many cases, heightened tension of the masticatory muscles exacerbates these symptoms, while parafunctional habits such as clenching or bruxism may further aggravate the condition [6]. The clinical presentation can range from mild discomfort to severe pain episodes, often accompanied by restricted mandibular mobility or episodes of joint hypermobility [7].

Given the diverse clinical presentations and underlying causes of TMDs, management typically involves a multidisciplinary approach integrating both non-invasive and invasive treatment modalities. Conservative treatment options include patient education, behavioral modification, physiotherapy and jaw exercises, massage therapy, acupuncture, occlusal splints, and pharmacotherapy such as non-steroidal anti-inflammatory drugs, muscle relaxants, and antidepressants. Physical therapeutic modalities frequently employed include ultrasound therapy, transcutaneous electrical nerve stimulation (TENS), and low-level laser therapy (LLLT). Psychological interventions may also be indicated in patients with associated stress-related or parafunctional habits. When conservative therapy fails, minimally invasive procedures such as trigger-point injections, intra-articular injections, and arthrocentesis can be considered, whereas surgical procedures, including arthroscopy and open joint surgery, are reserved for refractory cases [7]. Among non-invasive interventions, TENS and LLLT have gained significant attention for their therapeutic potential in pain relief and functional improvement [7-11].

The therapeutic efficacy of both modalities is highly dependent on treatment parameters. For LLLT, diode lasers in the 780-830 nm range, particularly at 810 nm with 0.2-0.5 W power and 2-6 J/cm² energy density, have been shown to reduce pain and improve mandibular function in TMD patients [7, 12]. Similarly, TENS outcomes are influenced by stimulation frequency, mode, and duration. Clinical trials have reported significant pain reduction using continuous stimulation at 50-100 Hz for 10-60 minutes, with electrodes placed over the masseter and temporalis muscle [13, 14].

TENS is widely used for neuromuscular pain modulation, aiming to reduce discomfort by stimulating nerve fibers and enhancing endorphin release. Meanwhile, LLLT has been recognised for its ability to promote tissue healing, reduce inflammation, and alleviate pain by modulating cellular activity.

Despite the established use of TENS and LLLT in TMD management, comparative studies evaluating their individual and combined effectiveness remain limited. The primary aim of this study is to conduct a comparative analysis to assess the efficacy of TENS and LLLT in reducing pain and improving pain-free mouth opening in patients with TMDs and whether LLLT is superior to TENS. Additionally, this study seeks to evaluate mid-treatment values using visual analog scale (VAS) scores and maximum mouth opening (MMO), providing novel insights into the progression of therapeutic outcomes.

## Materials and methods

This randomised clinical trial was conducted on patients clinically diagnosed with TMDs. The study was carried out in the Department of Oral Medicine and Radiology, Dr. R. Ahmed Dental College and Hospital, Kolkata, from November 2024 to October 2025. Ethical approval was obtained from the Institutional Ethics Committee, Dr. R. Ahmed Dental College and Hospital (Memo No: RADCH/EC/45/2024), and the trial was prospectively registered in the Clinical Trial Registry of India (CTRI/2024/07/071130). All participants provided written informed consent in accordance with the Helsinki Declaration.

The sample size was determined using G*Power software version 3.1.9.7 (Heinrich Heine University, Düsseldorf, Germany), based on a t-test model with an effect size of 0.98, an alpha error of 0.05, and a power of 80%, using a two-tailed significance level (α = 0.05). The effect size was estimated based on previous research [12]. This resulted in a required sample size of 36, with 18 patients per group. However, considering a 10% dropout rate during follow-up, the final sample size was inflated to 40 patients.

A total of 40 patients aged between 25 and 50 years were randomly selected from the outpatient department after obtaining informed consent. Patients were included if they had a history of TMD-related pain for at least three months, were not taking any antidepressant medications, and clinically and radiographically diagnosed as cases of TMDs categorised under axis I research diagnostic criteria, 2014 (DC/TMD) [15]. Patients with five or more missing posterior teeth (apart from third molars), parafunctional behaviours including clenching and bruxism, impacted third molars, degenerative joint diseases, cardiac pacemakers, cancer, or any serious systemic illness were excluded. Additionally, patients who refused to get therapy were not included.

**Figure 1:**
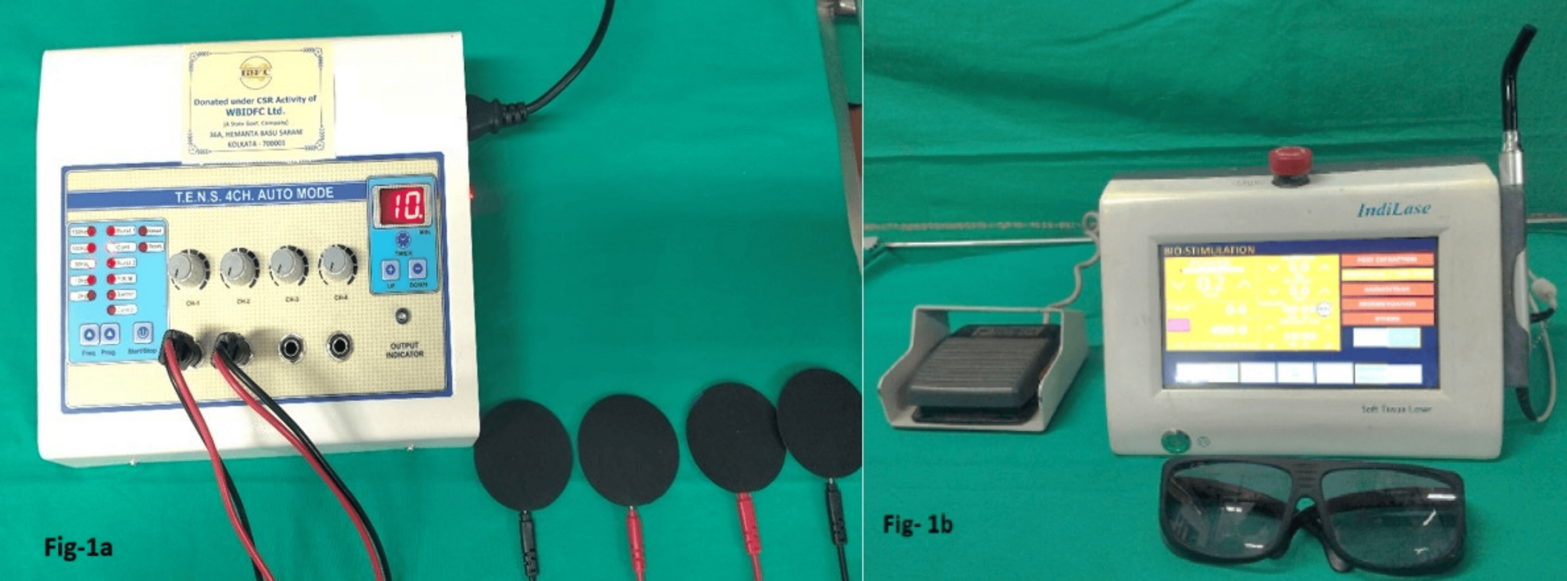
(a) WBIDFC TENS Machine (b) IndiLase diode laser WBIDFC TENS 4 CH device (Rebalance India, Ashok Nagar, New Delhi, India); IndiLase diode laser (Avalapalli Hudco, India).

Initially, 40 patients were randomly assigned to two groups (Group A and Group B) with 20 patients per group. An independent investigator, not associated with the study, used an online randomiser (https://www.random.org/) to generate the sequence. Group A was treated with TENS therapy for eight sessions (two sessions per week), using a WBIDFC TENS 4 CH device (Rebalance India, Ashok Nagar, New Delhi, India) (Figure 1a) with a frequency of 50 Hz, 200ms pulse width, in conventional mode for 10 minutes. For this, a four-electrode unit was placed on the bilateral pre-auricular region and masseter muscle region, with the intensity set according to the patient's tolerability. Group B underwent treatment with LLLT for eight sessions (two sessions per week), using an IndiLase diode laser (Avalapalli Hudco, India) (Figure 1b) in continuous wave mode with an 810 nm wavelength. A laser biostimulation tip of 0.3 cm^2^ was used to deliver a dosage of 6J/cm^2^ in a contact scanning motion over the tender/ trigger points.

Pain levels were recorded using a visual analogue scale (VAS) [14], where 0 represented no pain, and 10 represented the worst pain as subjectively perceived by the patient. Pain scores were recorded at baseline (prior to the first treatment session) and subsequently at each treatment visit immediately before the therapy session, resulting in eight serial measurements per participant.

Statistical analysis

After entering the data into Microsoft Excel 2021 (Microsoft Corp., Redmond, WA), GraphPad Prism for Windows, Version 10.1.2 (GraphPad Software, San Diego, CA), was used for analysis. The normality of data was verified using the Shapiro-Wilk test. Intra-group comparisons were carried out using repeated measures analysis of variance (ANOVA), whereas inter-group comparisons were done using an unpaired t-test. At P<0.05, significance was established.

## Results

Demographic characteristics

**Figure 2:**
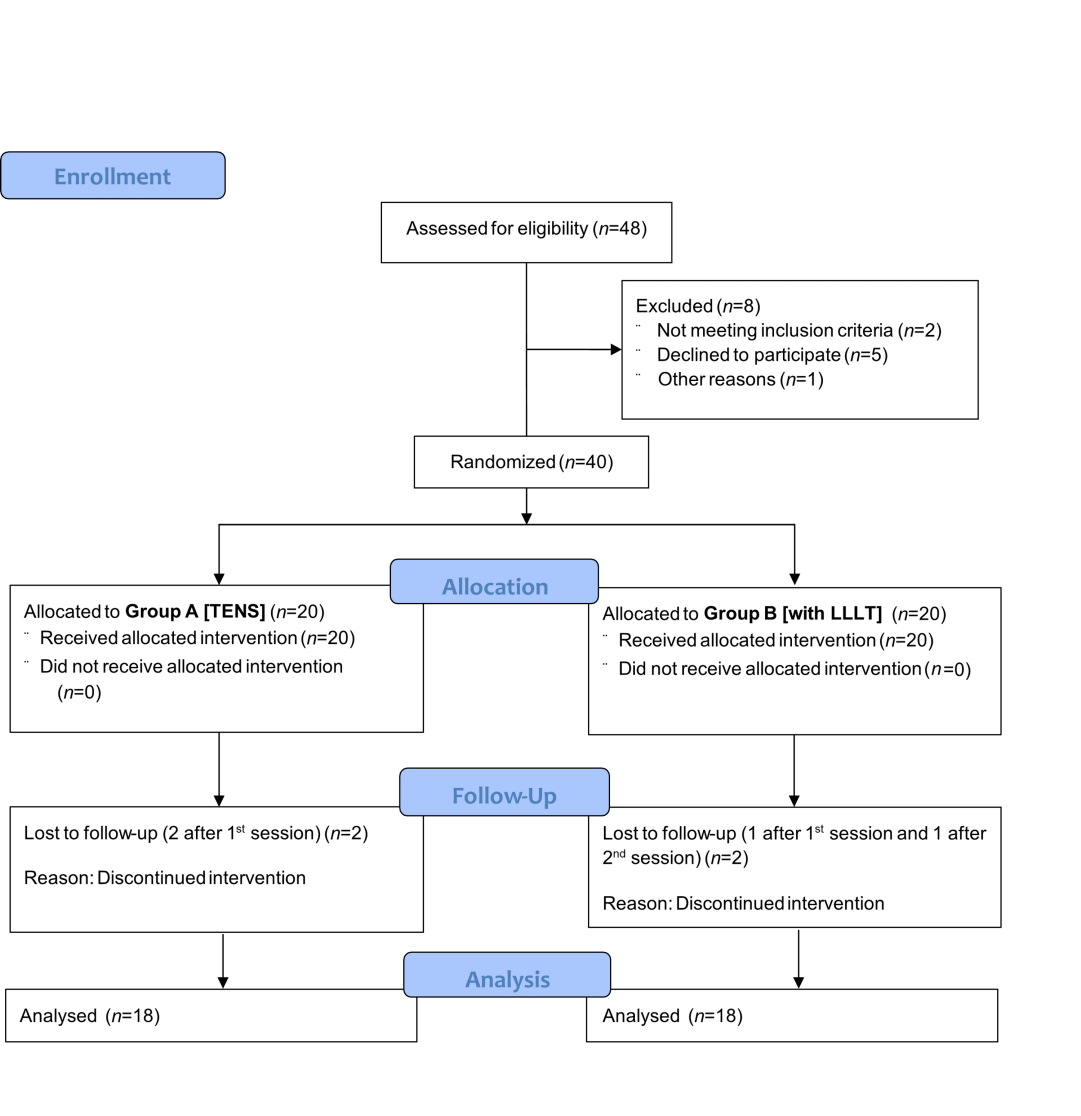
CONSORT flow diagram depicting participant enrollment, allocation, follow-up, and analysis Consolidated Standards of Reporting Trials (CONSORT) [16]

Two patients in each group were lost to follow-up in subsequent visits. Consequently, the final analysis included data from 18 patients per group (see Consolidated Standards of Reporting Trials (CONSORT) Flow Diagram in Figure 2). The demographic characteristics of the study subjects are presented in Table 1. The mean age of participants was 43.22 ± 14.49 years in the LLLT group and 38.61 ± 13.08 years in the TENS group, with no significant difference between them (P = 0.32). Gender distribution was also comparable, with female patients accounting for 77.8% in the LLLT group and 83.3% in the TENS group, while male patients comprised 22.2% and 16.7%, respectively (P = 0.67). These results indicate that age and gender were evenly distributed across the study groups, ensuring comparability (Table 1).

**Table 1:** Demographic characteristics of study subjects N: Total sample size; n: sample size per group a: analysed by chi-square test b: analysed by unpaired t-test *: Significant at P≤0.05; **: not significant (P>0.05)

Demographic characteristics		LLLT (n=18)	TENS (n=18)	Total (N=36)	Test statistic	P-value
Age (in years)^a^		43.22±14.49	38.61±13.08	40.9 ± 13.8	1	0.32^**^
Gender^b^	Female	14(77.8 %)	15(83.3 %)	29(80.6 %)	1.22	0.67^**^
	Male	4(22.2 %)	3(16.7 %)	7(19.4 %)		

Pain scores

**Figure 3:**
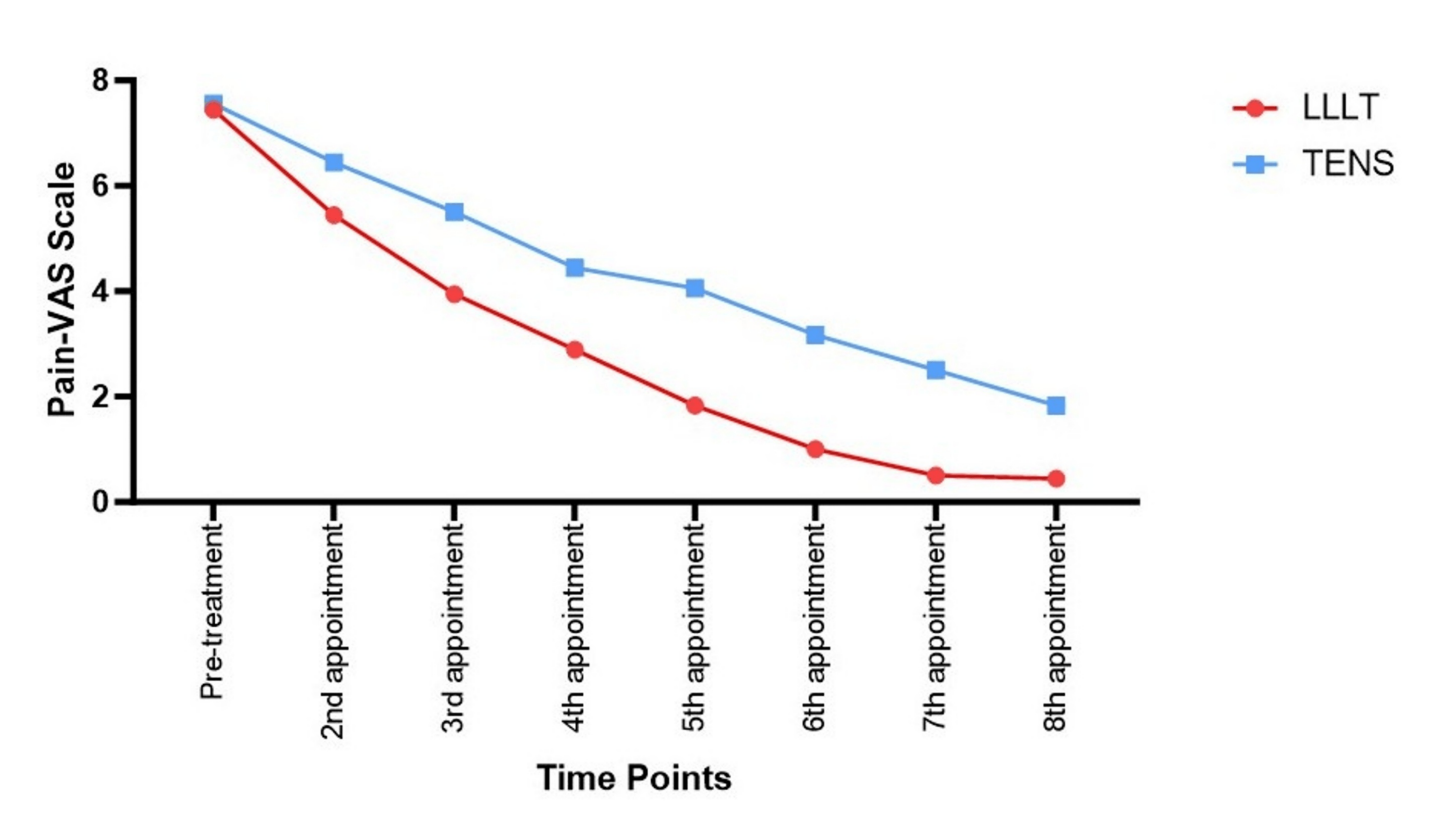
Line graph showing the trend of pain scores across time points for all the study groups LLLT: low-level laser therapy; TENS: transcutaneous electrical serve stimulation; VAS scale: visual analogue scale

A significant reduction in pain scores was observed from pre-treatment to subsequent appointments across both groups (Table 2, Figure 3). At baseline, pain scores were comparable (7.444 ± 1.58 in LLLT vs. 7.556 ± 1.688 in TENS). Over time, pain scores significantly decreased in both groups, with LLLT demonstrating a greater reduction at all time points. By the 8th appointment, mean pain scores had decreased to 0.444 ± 0.705 in LLLT and 1.833 ± 1.724 in TENS. Post-hoc comparisons showed that LLLT had a significantly greater reduction in pain compared to TENS at the 2nd appointment (P = 0.045), with differences persisting until the 8th appointment (P = 0.004). The most pronounced difference was observed at the 6th and 7th appointments, where LLLT exhibited significantly greater pain reduction (P < 0.001).

**Table 2:** Mean (± standard deviation) of the pain scores at all time points for all study groups LLLT: low-level laser therapy; TENS: transcutaneous electrical serve stimulation Δ Pre-op: Difference from the pre-treatment a: analysed by unpaired t-test; b: analysed by repeated measures ANOVA *: statistically significant

Time Points	LLLT (n=18)		TENS (n=18)		t-value^a^	P-value^a^ (LLLT vs UST)
	Pain scores	Δ Pre-op	Pain scores	ΔPre-op		
Pre-treatment	7.444±1.58	-	7.556±1.688	-	-	-
2nd appointment	5.444±1.338	2±1.645	6.444±1.79	1.111±0.676	2.12	0.045*
3rd appointment	3.944±0.938	3.5±1.383	5.5±1.948	2.056±0.802	3.83	-
4th appointment	2.889±1.278	4.556±1.854	4.444±2.148	3.111±1.079	2.86	0.008*
5th appointment	1.833±1.2	5.611±1.577	4.056±2.235	3.5±1.465	4.16	-
6th appointment	1±0.907	6.444±1.58	3.167±2.093	4.389±1.461	4.05	-
7th appointment	0.5±0.707	6.944±1.349	2.5±2.007	5.056±1.392	4.13	-
8th appointment	0.444±0.705	7±1.328	1.833±1.724	5.722±1.127	3.11	0.004*
F-value^b^	141.6	-	25.97	-	-	-
P-value^b^	<0.0001*	-	<0.0001*	-	-	-

Mouth opening

**Figure 4:**
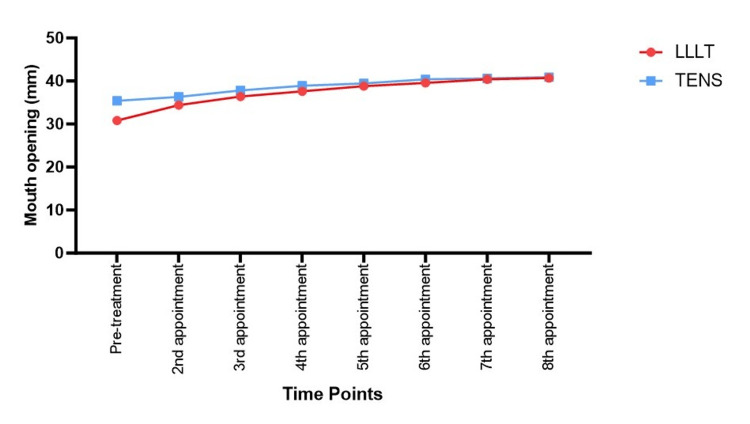
Line graph illustrating the mouth opening measurement trends across time points for all the study groups LLLT: low-level laser therapy; TENS: transcutaneous electrical serve stimulation

A significant increase in mouth opening (MO) was observed in both groups over time (Table 3, Figure 4). At baseline, MMO was 30.833 ± 7.771 mm in LLLT and 35.389 ± 7.868 mm in TENS. By the 8th appointment, MMO reached 40.667 ± 4.524 mm in LLLT and 40.889 ± 4.837 mm in TENS, indicating significant improvement in both groups. Pairwise comparisons indicated that LLLT had significantly greater improvement in MO compared to TENS from the 2nd appointment (P = 0.006), with differences persisting until the 8th appointment (P = 0.015). The most notable difference was observed at the 4th and 5th appointments, where LLLT showed a significantly greater increase in MMO (P = 0.018 and P = 0.012, respectively).

**Table 3:** Mean (± standard deviation) of mouth opening measurements at all time points for all study groups LLLT: low-level laser therapy; TENS: transcutaneous electrical serve stimulation Δ Pre-op: Difference from the pre-treatment a: analyzed by unpaired t-test, b: analyzed by repeated measures ANOVA *: statistically significant

Time Points	LLLT (n=18)		TENS (n=18)		t-value^a^	P-value^a^ (LLLT vs UST)
	MMO (mm)	Δ Pre-op	MMO (mm)	Δ Pre-op		
Pre-treatment	30.833±7.771	-	35.389±7.868		-	-
2nd appointment	34.389±6.06	3.556±3.434	36.333±7.364	0.944±1.259	3.03	0.006*
3rd appointment	36.389±5.71	5.556±4.693	37.833±6.767	2.444±1.977	2.59	0.016*
4th appointment	37.611±5.436	6.778±4.894	38.889±5.84	3.5±2.55	2.52	0.018*
5th appointment	38.833±5.205	8±5.391	39.444±6.022	4.056±3.152	2.68	0.012*
6th appointment	39.556±5.02	8.722±5.788	40.389±5.135	5±3.498	2.33	0.027*
7th appointment	40.389±4.654	18±40.389	40.611±4.852	18±40.611	2.61	0.014*
8th appointment	40.667±4.524	18±40.667	40.889±4.837	18±40.889	2.59	0.015*
F-value^b^	37.28	-	28.6	-	-	-
P-value^b^	<0.0001*	-	<0.0001*	-	-	-

## Discussion

The present study compared the effectiveness of LLLT and TENS in managing TMDs, revealing significant improvements in both pain reduction and maximum mouth opening over the treatment period [1]. The beneficial effects of LLLT on pain relief and tissue healing observed in our study are consistent with prior research demonstrating similar outcomes in TMD management [7]. TENS, on the other hand, is recognised for its ability to modulate neuromuscular pain through stimulation of nerve fibers and subsequent endogenous opioid release, supporting its effectiveness as observed here [5].

Epidemiological data indicate that TMD affects approximately 5-12% of the adult population and is more prevalent in women between 20 and 40 years of age, underscoring the clinical relevance of effective non-invasive treatment options [2,4]. In the present investigation, the LLLT group experienced a more pronounced reduction in pain scores at several mid-treatment appointments than the TENS group, which aligns with findings reporting superior pain reduction with LLLT [8, 17].

This trend is also supported by Buduru et al., who reported that LLLT significantly reduced TMD-related pain without any associated disadvantages, making it a recommended first-line treatment for these disorders [18]. Similarly, Del Vecchio et al. observed that LLLT effectively reduces TMD symptoms and improves overall jaw function [19]. Similarly, comparisons between LLLT and TENS have demonstrated a consistent trend favoring LLLT. A study by Dostalová et al. confirmed that LLLT significantly improved the range of motion in TMD patients while also facilitating substantial pain reduction [20]. Catão et al. and Sayed et al. further supported these findings, reporting notable improvements in pain control, increased mouth opening, fewer tender points, and a reduction in joint sounds following LLLT therapy [21, 22]. Moreover, Shobha et al. demonstrated that LLLT produced superior pain relief even after a one-month follow-up compared to placebo treatments, although they failed to notice any significant difference between the outcomes of LLLT and the placebo used in their study [23].

However, some studies have observed comparable efficacy between the two modalities, suggesting that differences in treatment parameters-such as variations in laser wavelength, power density, session duration for LLLT, and the frequency and mode settings for TENS-may influence outcomes [23]. Rezazadeh et al. (2017) examined 45 Iranian patients who did not respond to medications and found that both TENS and LLLT led to significant reductions in pain and tenderness, reinforcing their role in non-invasive TMD management [14]. Additionally, Seifi et al. conducted a study on 40 patients in Iran, concluding that both TENS and LLLT improved TMD symptoms, at least in the short term [24]. The multifactorial etiology of TMD, which includes variations in muscle tension and parafunctional habits, further highlights the potential for individual differences in treatment response [3].

Mechanistically, LLLT is believed to modulate cellular activity, reduce inflammation, and promote tissue repair--processes that collectively contribute to its effectiveness in reducing pain and enhancing joint function [7]. In contrast, TENS primarily functions by electrically stimulating peripheral nerve fibers to release endogenous opioids, offering effective analgesia but not directly influencing tissue repair [5]. Clinically, these findings suggest that both LLLT and TENS are effective non-invasive treatment options for TMD, with LLLT showing comparatively greater short-term improvement at several evaluated time points [11].

Recent meta-analyses (2020-2025) have reinforced these findings, consistently reporting superior short-term pain relief with LLLT compared to TENS. Ansari et al. and Ren et al. demonstrated that diode lasers, particularly at 810 nm, achieved significantly greater reductions in pain and muscle tenderness than TENS [25,26]. Díaz et al. further confirmed that LLLT enhances both pain control and mouth opening, with optimal outcomes observed at 810 nm wavelength and 2-6 J/cm² energy density [12]. Mechanistically, these differences can be explained by the distinct biological pathways: TENS primarily modulates nociceptive transmission via the Gate Control Theory, providing symptomatic analgesia without tissue repair, whereas LLLT induces mitochondrial photobiomodulation, enhancing ATP synthesis, reducing inflammation, and promoting tissue regeneration [27].

Both groups demonstrated significant improvement over time, indicating that TENS and LLLT are effective in the short-term management of TMD. However, the findings of the present study should be interpreted with caution due to the relatively small sample size and short follow-up duration, which may limit generalizability. Future studies with larger, multicentric populations and longer follow-up periods are warranted to confirm these findings and to explore the potential synergistic effects of combining TENS and LLLT.

## Conclusions

Both TENS and LLLT are useful non-invasive treatments for treating symptomatic TMDs, according to this randomised controlled experiment. But as compared to TENS, LLLT showed better pain relief and more MMO improvement, especially during mid-treatment and post-treatment evaluations. Within the limitations of the present study, LLLT demonstrated greater short-term improvement than TENS at several evaluated time points; however, these findings should be interpreted cautiously due to the limited sample size and short follow-up duration.

These findings contribute to the existing evidence supporting the role of LLLT as a non-invasive modality in the management of TMDs. Further well-designed studies with larger samples and longer follow-up are required before translating these findings into broader clinical recommendations.
